# Production of Defatted Palm Kernel Cake Protein Hydrolysate as a Valuable Source of Natural Antioxidants

**DOI:** 10.3390/ijms13078097

**Published:** 2012-06-29

**Authors:** Mohammad Zarei, Afshin Ebrahimpour, Azizah Abdul-Hamid, Farooq Anwar, Nazamid Saari

**Affiliations:** 1 Department of Food Science, Universiti Putra Malaysia, 43400 UPM, Serdang, Selangor, Malaysia; E-Mails: mzarei.mail@gmail.com (M.Z.); a_ebrahimpour@yahoo.com (A.E.); azizahhamid@mosti.gov.my (A.A.-H.); 2 Department of Chemistry, University of Sargodha, Sargodha, 40100, Pakistan; E-Mail: fqanwar@yahoo.com

**Keywords:** antioxidant, palm kernel cake, protein hydrolysates, HPLC, bioactive peptide

## Abstract

The aim of this study was to produce a valuable protein hydrolysate from palm kernel cake (PKC) for the development of natural antioxidants. Extracted PKC protein was hydrolyzed using different proteases (alcalase, chymotrypsin, papain, pepsin, trypsin, flavourzyme, and bromelain). Subsequently, antioxidant activity and degree of hydrolysis (DH) of each hydrolysate were evaluated using DPPH• radical scavenging activity and *O*-phthaldialdehyde spectrophotometric assay, respectively. The results revealed a strong correlation between DH and radical scavenging activity of the hydrolysates, where among these, protein hydrolysates produced by papain after 38 h hydrolysis exhibited the highest DH (91 ± 0.1%) and DPPH• radical scavenging activity (73.5 ± 0.25%) compared to the other hydrolysates. In addition, fractionation of the most effective (potent) hydrolysate by reverse phase high performance liquid chromatography indicated a direct association between hydrophobicity and radical scavenging activity of the hydrolysates. Isoelectric focusing tests also revealed that protein hydrolysates with basic and neutral isoelectric point (pI) have the highest radical scavenging activity, although few fractions in the acidic range also exhibited good antioxidant potential.

## 1. Introduction

Palm kernel cake (PKC), a main by-product of the palm oil extraction process, due to its poor utility, is mostly used for animal feed. It can be explored as a potential source of valuable components, especially the plant protein (the content ranging between 15–17%), for human nutrition. In Malaysia, PKC is produced in large quantities (around 2.2 million tons/annum) as a result of oil extraction from oil palm. This amount is significant enough to consider PKC as a bioresource of valuable components and raw materials, such as cellulose, hemicellulose, protein and lignin for several industries. At present, PKC is mostly used as a feed for pigs, aquaculture and ruminants [1]. Enzymatic hydrolysis is one of the possible ways to improve the value of PKC by adding useful functional properties and bioactivities, such as antioxidant function.

Antioxidants have a positive effect on health as they can protect the human body against different disorders caused by reactive oxygen species (ROS) [2]. Antioxidant-prooxidant balance also is one of the most important systems in the human body that can be changed with the progression of age and other factors, such as environmental pollutants, fatigue, excessive caloric intake, and high fat diets [3–5]. With advancing age, the plasma and cellular antioxidant potential as well as the absorption of nutrients, including antioxidants, gradually decreases [3]. This explains why aging is accompanied by the accumulation of oxidized compounds, for example, protein carbonyls [4]. In an “ideal” biological system and a clean environment within which to live, the body’s endogenous antioxidants provide an adequate health protection. Use of dietary antioxidants has been recognized as potentially effective to promote human health by increasing the body’s antioxidant load [5]. Currently, natural antioxidants have received considerable interest by the food and therapeutic industry due to consumer preference and concern over the safety of synthetic antioxidants [6]. Plant and animal proteins are well known as sources of bioactive peptides.

Meanwhile, the demand for the use of bioactive peptides and protein as antioxidant ingredients in food is rapidly increasing due to the low cost, safety and their inherent nutritional and functional value. Some bioactive peptides, such as carnosine, anserine, and glutathione are well-known for their endogenous antioxidative effects [7]. Several bioactive peptides have been isolated from different protein hydrolysates through the route of enzymatic proteolysis [8]. In addition, several amino acids, for example tyrosine, methionine, histidine, tryptophan and proline can act as antioxidants [9]. The antioxidant efficacy of protein hydrolysates and peptides depends on the source of protein, the protein substrate pretreatment, the type of proteases used, and the hydrolysis conditions applied. Since protein hydrolysate is a heterogeneous mixture that can contain both antioxidant and prooxidant peptides, it is desirable to fractionate the peptide mixture to remove oxidizing components [10].

In general, antioxidant peptides are capable of acting as radical scavengers, proton donors, and metal-ion chelators [5–10]. A number of multiple biological activities, including anticancer, anti-inflammatory [11], antioxidant [12], cholesterol-lowering [13] and blood pressure-lowering (ACE inhibitory) [14] have been ascribed to different biopeptides. Bioactive peptides are inactive within the sequence of parent protein and can be released during enzymatic hydrolysis of the protein. After releasing, they may exert various physiological functions.

To the best of our knowledge, there is no report on the bioactivity and functionality of PKC protein and protein hydrolysates. Therefore, the main aim of this study was to explore the functional biopeptide potential of PKC by hydrolysis of its protein and evaluating the antioxidant activity of its protein hydrolysates, based on their free radical scavenging activity.

## 2. Results and Discussion

### 2.1. Proximate Analysis

Proximate composition of the PKC is shown in Table 1. The level of main nutritional components, namely carbohydrates and protein, were found to be 50.4 and 17.6%, respectively. Based on literature, the contents of protein and other nutrients in different types of PKC depend on the variety of oil palm and the type of oil extraction process. In previous studies, the protein content in different types of PKC ranged from 15–21% [15].

### 2.2. Degree of Hydrolysis

The degree of hydrolysis (DH) measures the progress of hydrolysis of protein. DH has been defined as the percent ratio of the number of peptide bonds cleaved to the total number of peptide bonds in the substrate studied [16]. It is the proportion of cleaved peptide bonds in a protein hydrolysate [17].

DH is the most widely-used indicator for comparing different protein hydrolysates. During the hydrolysis, different varieties of peptides are generated, depending on enzyme specificity. Several methods are employed for determining the DH. The most commonly used methods include the pH-stat, trinitrobenzenesulfonic acid (TNBS), *O*-phthaldialdehyde (OPA), trichloroacetic acid, soluble nitrogen (SN-TCA), and formol titration methods. The pH-stat method is based on the number of protons released during hydrolysis; the TNBS, OPA, and formol titration methods are based on the measurement of amino groups generated from hydrolysis. The SN-TCA method measures the amount of TCA-soluble nitrogen, rather than DH. The pH-stat is the simplest and most commonly used method, but does not determine peptide bonds directly and it is not a buffering hydrolysis system. In addition, the accuracy of the method depends on the type of hydrolytic enzymes used, the size of the hydrolyzed peptides, and the reaction temperature [17].

Generally, the TNBS and OPA methods are comparable and directly determine the DH [17]. However, the TNBS method is laborious, and involves the use of hazardous and unstable chemicals, and therefore cannot be used to follow a hydrolysis reaction continuously. On the other hand, the OPA method is more accurate, easier, environmentally safer and faster, and has a broader application range as compared to the TNBS method [18].

In the current study, PKC protein extracted with NaOH solution and precipitated by HCl was hydrolyzed. The degree of hydrolysis was monitored and the antioxidant activity of the hydrolysates was determined by radical scavenging activity assay.

**Figure 1:**
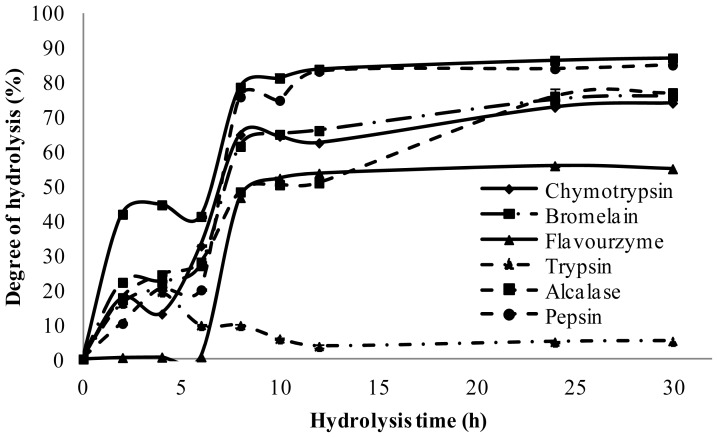
Time course of palm kernel cake protein hydrolysis with different enzymes.

**Figure 2:**
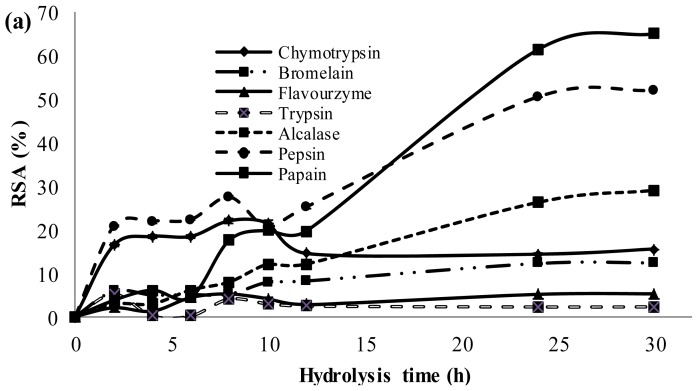

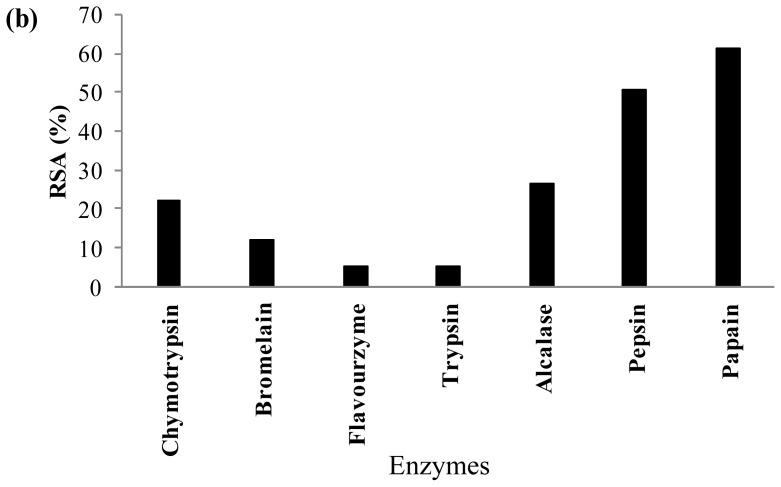
Radical scavenging activity (RSA) of palm kernel cake protein hydrolysates generated by different enzymes. (**a**) time course of RSA of hydrolysates generated by different proteases, and (**b**) maximum RSA of hydrolysates achieved by different proteases.

As shown in Figure 1, at the end of the hydrolysis reaction (30 h), the hydrolysates obtained with trypsin, flavourzyme, chymotrypsin, bromelain, alcalase, pepsin and papain exhibited significantly (*p* < 0.05) different DH of 19 ± 0.11%, 56 ± 0.15%, 74 ± 0.27%, 76 ± 1.3%, 77 ± 2.1%, 85 ± 0.23%, 87 ± 0.1%, respectively. A similar hydrolysis curve was investigated by Qin *et al.* [19], where, in their study, papain and pepsin had the highest DH while trypsin exhibited the lowest value. Figure 2 revealed that further incubation with all the enzymes caused a significant increase in DH. Based on the results, after 24 h hydrolysis, the DH for most of the enzymes reached the plateau, confirming that hydrolysis was completed.

All graphs show a high rate of hydrolysis after adding enzymes, followed by a decrease in the rate after two hours. However, papain resulted in the fastest increases in DH, followed by pepsin, bromelain, alcalase, chymotrypsin, trypsin and flavourzyme. This result was in accordance with the findings of Tang *et al.* [20], showing the fastest increase in hydrolysis with the alcalase and pepsin.

### 2.3. Antioxidant Activity of Palm Kernel Cake Protein Hydrolysates

In this research, radical scavenging activity (DPPH•) was employed to evaluate the antioxidant activity of PKC peptides. Free radicals are very reactive and they can stay in short time. DPPH• is one of the free radicals which can be stable even at room temperature. The DPPH• radical has a single electron and shows maximum absorbance at 517 nm. Its ethanolic solution exhibits crimson in color. The absorbance of ethanolic DPPH• solution at 517 nm reduces gradually while the free radicals are scavenged and the color of the solution changes from crimson to yellow [21]. The method is based on the reduction of the absorbance of ethanolic DPPH• solution at 517 nm in the presence of a proton-donating substance, and the formation of the diamagnetic molecule by accepting an electron or hydrogen radical [8,22].

After the proteases digestion, hydrolysates with high DH are expected to expose a more hydrophobic amino acid side chain, smaller size of protein hydrolysate and become more accessible by DPPH• radical scavenging activity [19–23].

**Figure 3:**
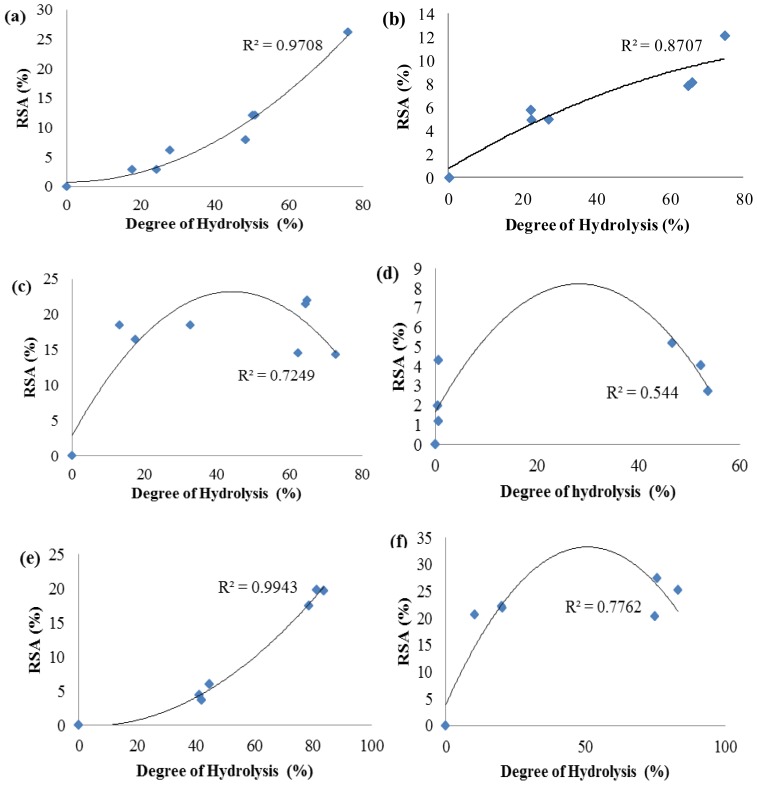

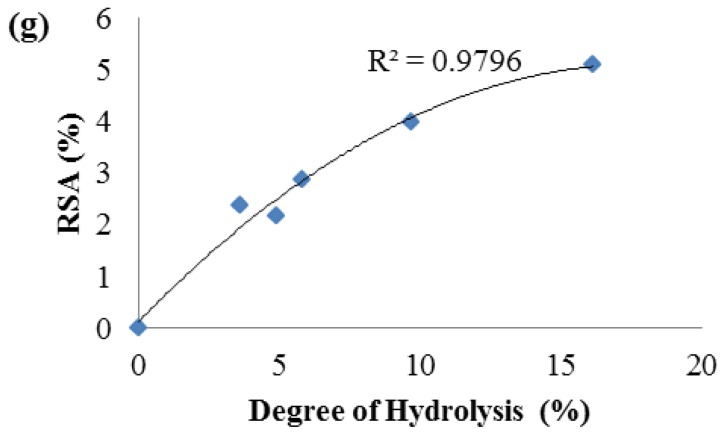
Function of degree of hydrolysis versus radical scavenging activity (RSA) of PKC protein hydrolysates produced by different enzymes: (**a**) Alcalase; (**b**) Bromelain; (**c**) Chymotrypsin; (**d**) Flavourzyme; (**e**) Papain; (**f**) Pepsin; (**g**) Trypsin.

Figure 2 shows the DPPH• radical scavenging activity of PKC protein hydrolysates generated with different proteases. The results indicated that protein hydrolysates obtained by treatment using papain from PKC protein exhibited the highest radical scavenging activity (65 ± 0.25%), while the lowest was obtained with trypsin (2.2 ± 0.29%). On the other hand, protein hydrolysates prepared with the pepsin, alcalase, chymotrypsin, bromelain, and flavourzyme exhibited 52%, 29%, 15.5%, 12.3% and 5.2% scavenging activity, respectively (Figure 2a). Figure 2b shows the highest antioxidant activity of PKC protein hydrolysates achieved by different proteases. As shown in Figure 3, the activity had reverse correlation with peptide size as supported by many previous studies, which have also reported that low MW peptides possessed strong DPPH• radical scavenging ability [19–24].

Figure 3 shows that in the cases of papain, bromelain, flavourzyme, and trypsin, increasing the DH, caused enhancement in radical scavenging activity. However, for alcalase, pepsin and chymotrypsin initially by increasing the DH, radical scavenging activity increased with a maximum activity at 10 h followed by a decrease in radical scavenging activity. This is in contrast with studies of Klompong *et al.* [25] and Jamdar *et al.* [26], who showed a decrease in DPPH• radical scavenging activity of yellow stripe trevelley protein hydrolysate with increase in the DH. On the other hand, these results are in accordance with some studies [19–27], which reveal an increase in DPPH• radical scavenging activity with the increase of DH. Therefore, increasing the DH of PKC protein might have produced peptides which are electron donors and can react with free radicals to convert them to more stable products and terminate the radical chain reaction, where low MW peptides compositions showed higher DPPH• radical scavenging activity [28].

In the present study, papain hydrolysate with 38 h hydrolysis time showed the highest activity (73.5%) among all PKC protein hydrolysates.

### 2.4. Characterization of Papain Hydrolysate

#### 2.4.1. Hydrophobicity

**Figure 4:**
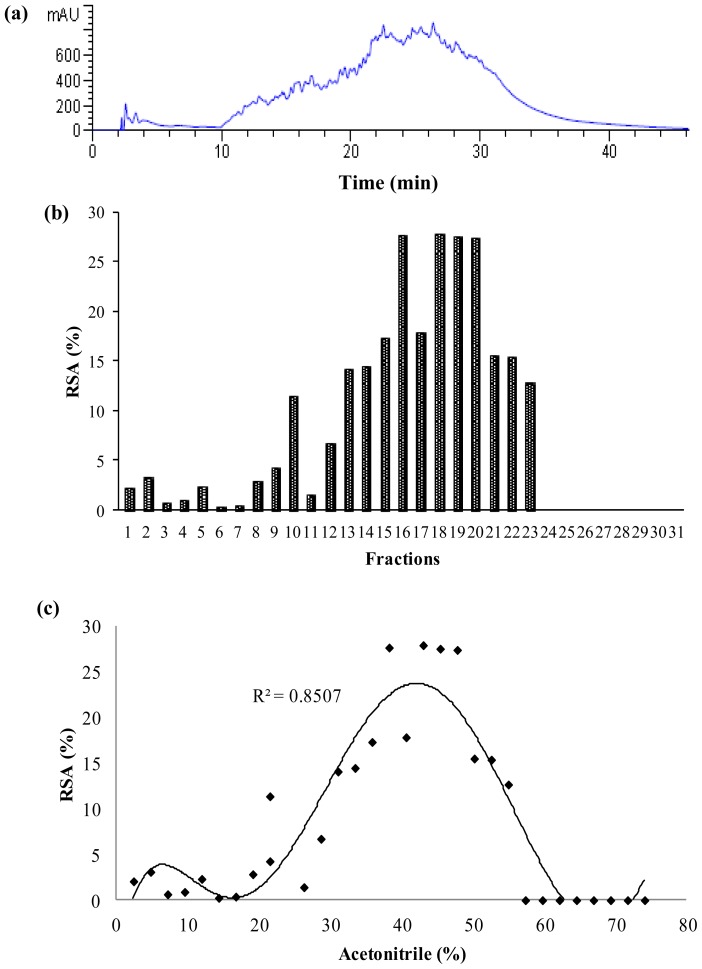
(**a**) Chromatogram of PKC protein hydrolysate separated by semi-preparative RP-HPLC; (**b**) Free radical scavenging activities of each fraction; (**c**) Function of RSA *versus* acetonitrile percentage used.

In order to study the hydrophobicity nature of the antioxidant peptides produced, RP-HPLC was employed. In reversed-phase HPLC, compounds are separated on the basis of their hydrophobic character; that is, peptides with large hydrophobicity values have longer elution times in a C-18 reversed phase column. In this study, because the papain hydrolysate exhibited higher antioxidant activity than the other enzymes hydrolysates, it was analyzed by RP-HPLC (Figure 4). Thirty-one fractions were collected, lyophilized, and their antioxidant activity was determined. Results revealed a meaningful relation between radical scavenging activity and hydrophobicity (Figure 4c).

Fractions 16, 18, 19 and 20 showed relatively strong hydrophobicity with the greatest radical scavenging activity for DPPH•, where their radical scavenging activities were 28%, 28%, 28% and 27%, respectively, while they were diluted 80-fold in comparison with the initial papain hydrolysate (before RP-HPLC). Therefore, the results demonstrated that the free radical scavenging activity of PKC protein hydrolysate correlated not only to the size and degree of hydrolysis but also to the hydrophobicity of peptides. This result was in accordance to some previous studies [5,6,29]. Since fractions 18, 19 and 20 showed the highest DPPH• radical scavenging activity, they were selected for further characterization, based on isoelectric point.

#### 2.4.2. Isoelectric Properties of Hydrolysates Peptides Produced

In order to assess the isoelectric properties of the antioxidant peptides, the isoelectric focusing electrophoresis using OFFGEL system (Agilent Technology, Waldbronn, Baden-Wuerttemberg, Germany) was employed. According to isoelectric focusing electrophoresis, protein or peptide separation takes place in a two-phase system, with an upper liquid phase that is divided into compartments and a lower phase being a conventional rehydrated IPG gel strip. Typically, the sample is diluted into the focusing buffer and loaded into all wells. Because there is no fluidic connection between the wells, proteins or peptides are forced to migrate through the IPG gel where the actual separation takes place. After isoelectric focusing (IEF), the proteins or peptides are present in the liquid phase and can be recovered conveniently from the wells for further processing. In this fractionator, proteins or peptides are separated according to their isoelectric point (pI) in a multi-well device with the advantage of being directly recovered in solution for further analysis. For pI-based peptide separation, the Agilent 3100 OFFGEL Fractionator with a 12-well setup and a 1 cm, pH 3–10 IPG strip was used according to the protocol. The sample was focused with a maximum current of 50 μA and typical voltages ranging from 500 V to 4000 V, until 50 kVh was reached after 24 h.

**Figure 5:**
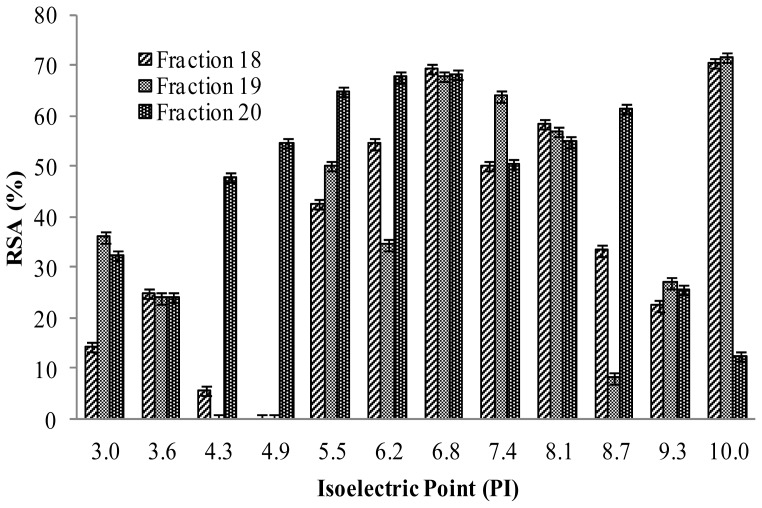
DPPH• radical-scavenging activity of papain protein hydrolysate fractions with different isoelectric points.

Figure 5 shows the DPPH• radical-scavenging activity of OFFGEL fractions of PKC protein hydrolysate obtained from OFFGEL fractionator. The DPPH• radical-scavenging activity was higher in fractions with pI 5–7 and fractions 18 and 19 with pI 10 compared to other isoelectric points. The highest activity was 71.7% and 70.5% for fraction 18 and 19, respectively (pI = 10), followed by 69.3%, 67.7% and 68.3% for fractions 18, 19 and 20 (pI = 7). The results presented here are in contrast with the results of Park *et al*. [30] where they reported acidic fractions (pI < 6) have the highest DPPH• radical scavenging activity, and is also in accordance with the results of Tsuge *et al*. [31] and Chen *et al*. [6], But in this study, high antioxidant activity was exhibited by fractions with pI = 5–7 and pI = 10. Thus the fractions at pI = 7 and pI = 10 are in contrast with the results reported by Park *et al*. [30].

## 3. Experimental Section

### 3.1. Materials

Palm kernel cake (PKC) used in this study was obtained from the My-4-Season’s company, Serdang, Malaysia. Alcalase was obtained from Novoenzyme Co. (Nottingham, UK). Pepsin, flavourzyme, *O*-phtaldialdehyde (OPA) and 1, 1-diphenyl-2-picrylhydrazyl (DPPH• radical) were purchased from Sigma Aldrich (Munich, Germany). Papain, bromelaine and glutathione were from Acros Organics Co. (St. Louis, MO, USA). Trypsin was obtained from Fisher Scientific Co. (Fair Lawn, NG, USA). Chymotrypsin was purchased from Calbiochem, South Africa. Ethanol, acetic acid, sodium acetate, potassium phosphate, Tris-base and potassium chloride were obtained from Merck Co. (Darmstadt, Germany).

### 3.2. Preparation of Palm Kernel Cake Protein Isolate

Palm kernel cake (PKC) protein isolate was produced according to the method as described by Arifin *et al*. [32] with minor modification. Briefly, PKC was defatted with petroleum ether, and solvent was removed by rotary evaporator. The defatted PKC was dried in the ventilator overnight at 20 °C. PKC protein isolate was obtained by dispersing the defatted PKC in NaOH solution (0.03 N) at ratio 1:30 (w/v) and extracted by stirring for 2 h using a water bath shaker. After filtration, the pH of the supernatant was adjusted to 3.5 (isoelectric point of PKC protein), the precipitate was obtained by centrifugation at 10,000 × g for 10 min and stored at −80 °C, until used for further analysis.

### 3.3. Preparation of PKC Protein Hydrolysates

PKC protein hydrolysates were prepared according to the method as described by Hwang *et al*. [33]. PKC protein (0.65 g) were well mixed with papain (65 °C, pH 6.5), alcalase (55 °C, pH 7.5), pepsin (37 °C, pH 1.5), trypsin (37 °C, pH 8), flavourzyme (55 °C, pH 8), bromelain (55 °C, pH 5), chymotrypsin (50 °C pH 6.8) at a ratio of 50:1 (w/w) in 35 mL of buffer solution and incubated in a water bath shaker at optimal conditions (Table 2). The reactions were stopped by heating the mixture in 100 °C boiling water for 10 min. After centrifugation (10,000 × *g*, 4 °C, 10 min) samples were prepared for radical scavenging activity assays. Hydrolysis time was 30 h for all enzymes. Each enzyme was individually added to PKC protein after every 6 h at 1:50 ratio for the first 12 h, the enzymatic hydrolysis was continued for another 12 h without adding the enzyme and also another 6 h with adding enzyme.

Previous studies have shown that hydrolysis time lower than 6 h resulted in a degree of hydrolysis less than 50%. Therefore, in the present study, enzymes were added at 4 different times (0, 6 and 12 and 24 h) to increase the degree of hydrolysis, and for the production of smaller peptides. Figure 1 presents the time course study of DH. Adding enzyme at different times might produce different protein sequences probably with different antioxidant activity.

### 3.4. 1,1-Diphenyl-2-Picrylhydrazyl Free Radical Scavenging Assay

The measurement of 1,1-diphenyl-2-picrylhydrazyl (DPPH•) free radical scavenging activity was done according to the method described by Hwang *et al*. [33] with little modification. After mixing 250 μL hydrolysate aliquot with 250 μL buffer solution and 500 μL 0.15 mM DPPH• (in 80% ethanol), the mixture was kept in the dark at room temperature (25 °C) for 45 min and absorption at 517 nm was measured. The scavenging activity was expressed as shown in the following equation:

$$\begin{array}{c} \text{DPPH}•\text{radical scavenging activity }(\%)=\\ {}[(\text{Blank absorbance}-\text{Sample absorbance})/\text{Blank}\;\text{absorbance}]\times 100 \end{array}$$

### 3.5. *O*-Phthaldialdehyde Spectrophotometric Assay

A rapid, sensitive and convenient *O*-phthaldialdehyde (OPA)-based spectroscopic assay was performed to measure proteolysis of PKC proteins in buffered solutions according to the method of Church *et al.* [34] and Salami *et al.* [35] with minor modifications. Fresh OPA solution was prepared daily as follows: 7.62 g sodium tetra hydroborate and 200 mg of SDS dissolved in 150 mL deionized water were mixed with OPA solution (160 mg in 4 mL of ethanol 96%). This solution was mixed with 50 mL of deionized water containing 400 μL β-mercaptoethanol. Proteolysis assay was performed as follows: 36 μL of the sample was added directly to 270 μL of OPA reagent in a 96-well plate assembly. The solutions were incubated for 2 min at room temperature, and then the absorbance at 340 nm was measured using an ELISA Reader (Labomed, model UVD-2950, Culver City, CA, USA).

### 3.6. Characterization of PKC Protein Hydrolysates

#### 3.6.1. Reverse Phase High Performance Liquid Chromatography

The PKC protein hydrolysate produced by papain was loaded into a semi-preparative C18 RP-HPLC column (9.40 mm × 250 mm, 5-μm particles, Agilent Technologies, Santa Clara, CA, USA). The sample injection volume and concentration were 250 μL and 0.2 mg of peptide per mL, respectively. The column was eluted using eluent A, deionized water containing 0.1% (v/v) TFA and eluent B, acetonitrile containing 0.1% (v/v) TFA. Elution was carried out according to the following process: 0–5 min, 100% eluent A; 5–60 min, 0–100% eluent B. The flow rate was 4 mL per min and the detection wavelength 205 nm. All fractions were collected and freeze-dried. Each fraction, dissolved in 300 μL phosphate buffer, was analyzed for radical scavenging activity. The fractions with the highest antioxidant activity, obtained by semi-preparative reverse-phase high-performance liquid chromatography (RP-HPLC), was further purified, based on isoelectric focusing using the Agilent 3100 OFFGEL fractionator.

#### 3.6.2. Isoelectric Point Focusing Fractionation

To perform peptide fractionation according to their isoelectric point (pI), the 3100 OFFGEL fractionator and the OFFGEL Kit 3–10 (both from Agilent Technology, Waldbronn, Baden-Wuerttemberg, Germany) were used, following the user protocol. The device was set up for the three fractions separation by using a 24-cm-long IPG gel strip with a linear pH gradient ranging at 3–10. The peptides were separated in a two-phase system: liquid upper phase (focusing buffer provided by the supplier) separated in wells and lower IPG gel strip phase. There was no direct fluidic connection between the wells. The peptides migrated through the IPG gel that played the role of “bridge” between each well and were retrieved in the solution at the IPG region where pH was peptides pI.

40 μL of IPG Strip Rehydration Solution was added into each of the wells. It was incubated for 15 min without voltage to re-swell of the gel. After that, 150 μL of prepared (Protein or Peptide) OFFGEL sample (OFFGEL stock solution + Sample) was added into each well:

12 cm strip/12 well frame:150 μL×12 wells=1800 μL

To determine the antioxidant activity of fractions, a 50 μL portion from each fraction was suspended with 50 μL OFFGEL buffer, 50 μL phosphate buffer and 100 μL DPPH• solution to a final volume of 250 μL, then after 45 min being kept in the dark, the absorbance of each sample was measured at 517 nm using an ELISA Reader system (Power Wave × 340).

## 4. Statistics Analysis

All tests were performed in triplicate. Data were presented as means of three separate determinations and subjected to one-way analysis of variance using Statistical Analysis System Software (*SAS*, version 9.2; SAS institute: Cary, NC, USA, 2009). Significant differences between mean values were determined by Duncan’s multiple range tests and accepted at *p* < 0.05.

## 5. Conclusions

Malaysia is one of the most important producers of PKC in the world. To enhance the added value of PKC, its protein was hydrolyzed to generate bioactive peptides. In this study, PKC protein hydrolysates obtained using various proteases were found to possess antioxidant activity. Among seven proteolytic preparations, protein hydrolyzed by papain, resulted in the production of the hydrolysate with the highest antioxidant activity. Based on the results, smaller and more hydrophobic peptides with basic or neutral pI showed higher antioxidant activity.

## Figures and Tables

**Table 1:** Proximate compositions of palm kernel cake.

Parameters	Amount (%)
Protein	17.6 ± 1.4
Carbohydrates	50.4 ± 2.3
Crude fat	5.5 ± 0.3
Crude fiber	11.5 ± 1.3
Moisture	8.9 ± 1.1
Ash	6.1 ± 1.2

**Table 2:** Conditions for the hydrolysis of PKC protein with different enzymes.

Enzyme	Buffer (50 mM)	pH	Temperature (°C)	Enzyme to Substrate Ratio	Agitation Rate (rpm)
Pepsin	KCl-HCl	1.5	37	1:50	150
Papain	Phosphate	6.5	65	1:50	150
Bromelain	Acetate buffer	5.0	55	1:50	150
Alcalase	Phosphate	7.5	55	1:50	150
Chymotrypsin	Phosphate	6.8	50	1:50	150
Trypsin	Tris-HCl	8.0	37	1:50	150
Flavourzyme	Tris-HCl	8.0	55	1:50	150
